# Radioimmunotherapy for Malignant Mesothelioma Targeting C-ERC/Mesothelin

**DOI:** 10.3390/ph19030501

**Published:** 2026-03-18

**Authors:** Hirofumi Hanaoka, Aiko Yamaguchi, Masahiro Maeda, Tatsuya Segawa, Noboru Oriuchi

**Affiliations:** 1 Graduate School of Medicine, Gunma University, 3-39-22 Showa-machi, Maebashi 371-8511, Japan; 2 Near InfraRed Photo-ImmunoTherapy Research Institute, Kansai Medical University, 2-5-1 Shin-machi, Hirakata 573-1010, Japan; 3 Radiopharmaceuticals for Advanced Diagnostic Imaging and Therapy R&D Platform, Therapeutics Discovery Division, The University of Texas MD Anderson Cancer Center, 1881 East Rd., Houston, TX 77054, USA; 4 Immuno-Biological Laboratories Co, Ltd., 1091-1 Naka Aza-Higashida, Fujioka 375-0005, Japan; 5 Advanced Clinical Research Center, Fukushima Global Medical Science Center, Fukushima Medical University, Fukushima 960-1295, Japan; oriuchi@fmu.ac.jp; 6 Jyoban Hospital of Tokiwa Foundation, Kaminodai 57, Jyoban Kamiyunagaya, Iwaki 972-8322, Japan

**Keywords:** malignant mesothelioma, C-ERC/mesothelin, radioimmunotherapy

## Abstract

**Background/Objectives**: Malignant mesothelioma has a poor prognosis and limited therapeutic options. C-ERC/mesothelin is highly expressed in mesotheliomas and is a potential target for radioimmunotherapy (RIT). This study evaluated the radiolabeled anti-C-ERC/mesothelin antibody mAb 22A31 as a therapeutic agent. **Methods**: C-ERC/mesothelin expression in mesothelioma cell lines was assessed by Western blotting, and the specific binding of ^125^I-labeled mAb 22A31 was examined. Biodistribution of ^111^In-labeled mAb 22A31 was evaluated in a mesothelioma cell line, MSTO-211H tumor-bearing mice. The therapeutic efficacy of ^90^Y-labeled mAb 22A31 was evaluated in subcutaneous and pleural dissemination models. **Results**: mAb 22A31 showed specific binding considering the level of C-ERC/mesothelin expression in each mesothelioma cell line. ^111^In-mAb 22A31 accumulated in tumors with minimal uptake in normal tissues. ^90^Y-mAb 22A31 significantly delayed the growth of subcutaneous tumors and improved survival in a pleural dissemination model. **Conclusions**: Radiolabeled mAb 22A31 specifically targeted C-ERC/mesothelin and demonstrated therapeutic efficacy in a mesothelioma xerograph model. Therefore, ^90^Y-mAb 22A31 is a promising RIT agent and supports the further development of C-ERC/mesothelin-targeted therapy for mesothelioma.

## 1. Introduction

Malignant mesothelioma is an aggressive tumor with a poor prognosis and is classified into three main types based on histological features: epithelioid, sarcomatoid, and mixed [1,2]. The three therapeutic modalities currently in use, surgery, radiotherapy, and chemotherapy, do not offer a favorable prognosis, with a median survival time of 4–12 months. Most patients with malignant mesothelioma undergo chemotherapy; however, the disease is often diagnosed at an advanced stage [3,4]. Although newly developed molecular targeted drugs have improved patient prognosis, their therapeutic outcomes are still far from satisfactory [5]. Therefore, new treatments based on novel mechanisms are required.

Radioimmunotherapy (RIT), which uses radiolabeled monoclonal antibodies against molecules expressed on tumors, is a type of radionuclide therapy. RIT has been used for over 20 years for the treatment of non-Hodgkin’s lymphoma because anti-CD20 antibodies show high and specific accumulation in the lymphoma [6,7]. Because RIT is a systemic treatment, it is also effective for metastatic cancer, such as mesothelioma, which tends to spread diffusely [8]. Therefore, by selecting molecules specifically expressed in mesothelioma, RIT using antibodies against these molecules could be a potential therapeutic option.

The ERC⁄mesothelin gene encodes a 71 kDa precursor protein, which is cleaved by proteases into 31 kDa N-terminal (N-ERC/mesothelin) and 40 kDa C-terminal (C-ERC/mesothelin) fragments [9]. N-ERC/mesothelin, originally identified as megakaryocyte-potentiating factor, is a soluble protein that is released into the extracellular space and is now used as a serum marker of mesothelioma [10]. C-ERC/mesothelin is a glycoprotein anchored to the cell surface via a glycosylphosphatidylinositol and has been reported to promote anchorage-independent growth and prevent anoikis [11]. C-ERCs/mesothelin are highly expressed in malignant cells, such as mesotheliomas, pancreatic ductal carcinomas, ovarian cancers, and other cancers [12,13]. C-ERC/mesothelin is a promising therapeutic target [14], and the development of antibody-based therapy, such as antibody–drug conjugate (ADC) or chimeric antigen receptor T cells (CAR-T), is being developed. However, clinical treatment outcomes have been disappointing [14,15], and to our knowledge, no approved treatments targeting C-ERC/mesothelin exist. Nevertheless, mesothelin is still an attractive therapeutic target and could serve as a good target for RIT against mesothelioma since it is highly expressed in mesothelioma.

In this study, an anti-C-ERC/mesothelin antibody, mAb 22A31, was radiolabeled, and its binding affinity to C-ERC/mesothelin-positive tumor cells was assessed. Biodistribution studies were performed in tumor-bearing mice. The therapeutic efficacy of yttrium-90 (^90^Y)-labeled mAb 22A31 was evaluated in flank and disseminated tumor mouse models. Based on these results, the potential of ^90^Y-mAb 22A31 for mesothelioma was evaluated.

## 2. Results

### 2.1. C-ERC/Mesothelin Expression and Antibody Binding

**Figure 1:**
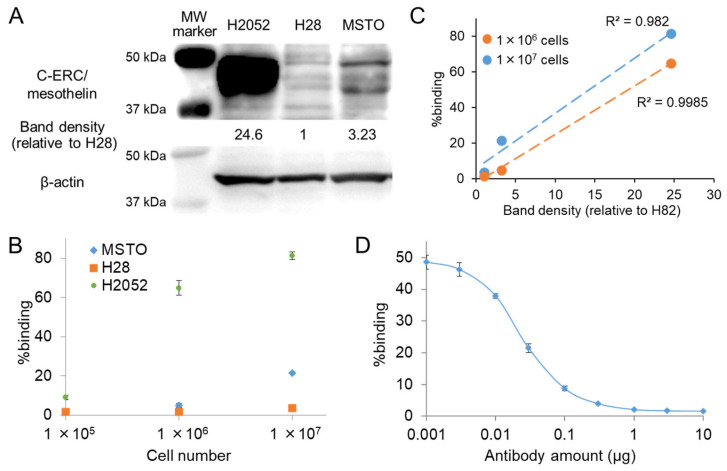
C-ERC/mesothelin expression and antibody binding. (**A**) The expression of C-ERC/mesothelin in three mesothelioma cell lines. Densitometric intensities of C-ERC/mesothelin are presented as folds relative to H28 band density (1.0). (**B**) Binding of ^125^I-mAb 22A31 to three cell lines at different cell numbers. (**C**) Correlation between the expression level of C-ERC/mesothelin and %binding of ^125^I-mAb 22A31 in three mesothelioma cell lines at different cell numbers. (**D**) Binding of ^125^I-mAb 22A31 to H2052 cells with various amounts of nonlabeled mAb 22A31. MW: molecular weigt, H2052: NCI-H2052, H28: NCI-H28, MSTO: MSTO-211H.

Western blotting analysis revealed that the expression levels of C-ERC/mesothelin differed significantly among the three mesothelioma cell lines (Figure 1A). Semi-quantitative analysis of C-ERC/mesothelin expression showed that NCI-H2052 cells exhibited very high expression (C-ERC/mesothelin band density of 24.6, relative to C-ERC/mesothelin-null NCI-H28), whereas MSTO-211H cells showed modest expression levels of 3.23.

^125^I-mAb 22A31 bound to each cell line in a cell number-dependent manner (Figure 1B). The extent of ^125^I-mAb 22A31 binding significantly correlated with the expression levels of the target molecule C-ERC/mesothelin in the three cell lines (Figure 1C, R^2^ > 0.95). The binding of ^125^I-mAb 22A31 to NCI-H2052 cells was inhibited by nonlabeled mAb 22A31 in a concentration-dependent manner (Figure 1D), demonstrating high-affinity and target-specific binding of mAb 22A31 to NCI-H2052 cells. The Kd value of mAb 22A31 was calculated to be 2.67 × 10^−10^ M, and the C-ERC/mesothelin expression level of NCI-H2052 cells was calculated to be approximately 90,000 molecules per cell using Scatchard plot analysis (R^2^ = 0.938).

### 2.2. Biodistribution Study

**Figure 2:**
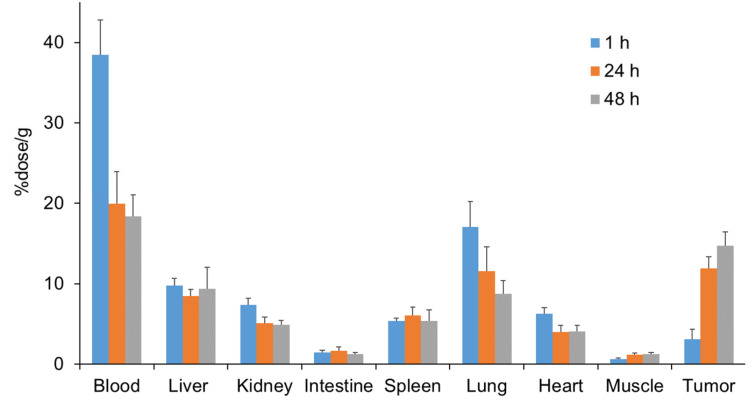
Biodistribution of radioactivity after injection of ^111^In-mAb 22A31 in MSTO-211H tumor-bearing mice. Each value represents the mean ± SD of five mice.

^111^In-mAb 22A31 accumulated in MSTO-211H tumors and was retained in tumors for at least 48 h post-injection at therapeutically relevant levels (Figure 2). The biodistribution of ^111^In-mAb 22A31 in the body was similar to that of conventional antibodies, indicating that it accumulated in the liver as an excretory organ. No specific retention of radioactivity was observed in organs other than the tumor. The tumor-to-blood ratio and the tumor-to-liver ratio were increased with time (tumor-to-blood ratio: 0.08 ± 0.03, 0.61 ± 0.08, and 0.81 ± 0.13 at 1, 24, and 48 h, respectively; tumor-to-liver ratio: 0.32 ± 0.13, 1.43 ± 0.28, and 1.66 ± 0.44 at 1, 24, and 48 h, respectively).

### 2.3. Therapeutic Study

**Figure 3:**
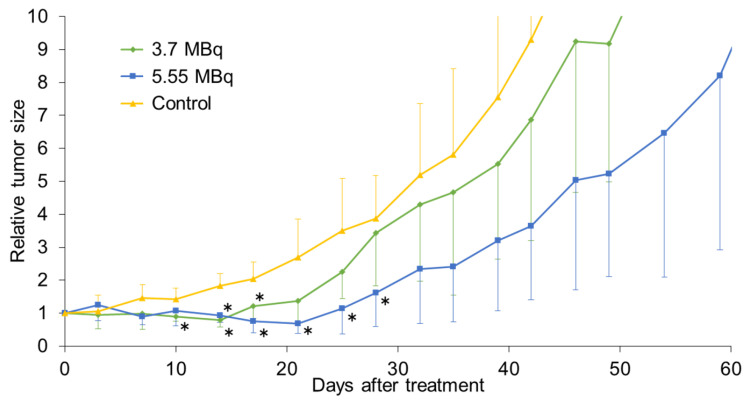
Tumor growth inhibition by RIT with ^90^Y-mAb 22A31 in subcutaneous tumor-bearing mice of MSTO-211H cells. Mice were intravenously administered with 3.7 and 5.55 MBq of ^90^Y-mAb 22A31. Untreated mice were used as controls. Each value represents the mean ± SD of four mice. A statistically significant difference from the control is indicated by * (*p* < 0.05).

In the subcutaneous model, tumor growth was significantly delayed in both ^90^Y-mAb 22A31 treatment groups compared to that in the control group (Figure 3). In addition, a temporary reduction in tumor size was observed in all treated mice at both doses. When comparing doses, the higher dose resulted in longer tumor growth suppression. Weight loss of just under 20% was observed in one of the four mice administered 5.55 MBq of ^90^Y-mAb 22A31, which subsequently recovered. No signs of radiation-induced toxicity were observed in the other mice.

**Figure 4:**
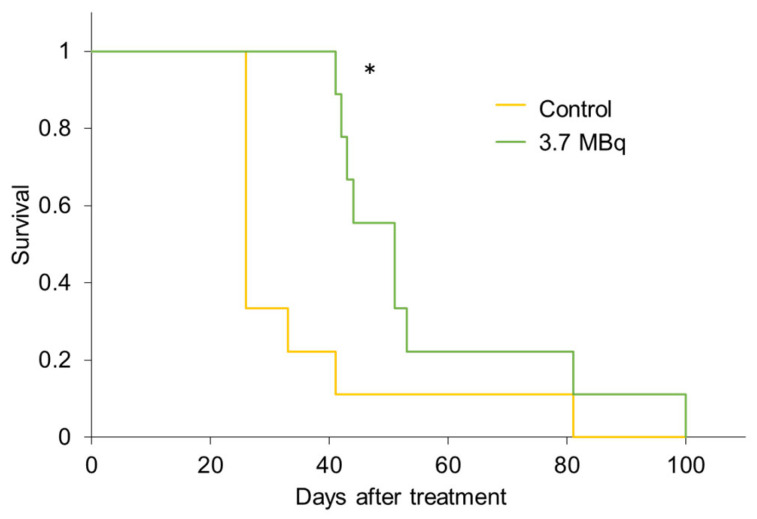
Kaplan–Meier survival curve analysis of the RIT with ^90^Y-mAb 22A31 in pleural seeding tumor-bearing mice of MSTO-211H cells. Mice were intrathoracically administered with 3.7 MBq of ^90^Y-mAb 22A31, and untreated mice were used as controls (nine mice in each group). A statistically significant difference from the control is indicated by * (*p* < 0.05).

In the peritoneal seeding model, Kaplan–Meier survival analysis showed that the survival of mice significantly improved with ^90^Y-mAb 22A31 treatment (Figure 4, *p* < 0.05). The median survival periods of the treatment and untreated groups were 51 days (95% confidence interval: 42.8 to 69.6) and 26 days (95% confidence interval: 22.7 to 46.4), respectively (χ^2^ = 5.701). More than half of the mice in the untreated group died by day 30, whereas no mice in the treatment group died by day 40. As all the mice died after a certain period, a tumor-bearing model would be successfully established. In addition, pleural dissemination was verified by dissecting several mice 4 weeks after the administration of MSTO-211H tumor cells (Supplementary Figure S1).

## 3. Discussion

C-ERC/mesothelin is an attractive target for RIT in mesotheliomas. This study evaluated the radiolabeled anti-C-ERC/mesothelin antibody, mAb 22A31, both in vitro and in vivo. mAb 22A31 was established by Ishikawa et al. [16] as a monoclonal antibody specific to C-ERC/mesothelin. ^125^I-labeled mAb 22A31 demonstrated sufficient binding to cells expressing C-ERC/mesothelin, and this binding was inhibited by nonlabeled antibodies, suggesting specific binding. Therefore, mAb 22A31 is a potential antibody for C-ERC/mesothelin-targeting therapy.

^111^In-mAb 22A31 accumulated in mesothelin-expressing tumors in vivo. NCI-H2052 cells with high C-ERC/mesothelin expression did not form measurable tumors in mice under the experimental conditions; therefore, tumor-bearing mice transplanted with MSTO-211H cells that expressed C-ERC/mesothelin at lower levels were used. Nevertheless, ^111^In-mAb 22A31 accumulated sufficiently in the MSTO-211H tumor. Therefore, in clinical settings, the presence of a certain degree of C-ERC/mesothelin expression, as in NCI-H2052 cells, is expected to result in higher antibody accumulation and considerable antitumor efficacy. No significant accumulation was observed in organs other than the tumors. Accumulation in the liver is observed during the general excretion process of antibodies. But the usual level of hepatic accumulation associated with excretion causes no significant adverse effects in the clinical RIT of malignant lymphoma [7]. Although further investigation in humans is required because mesothelin is minimally expressed in normal tissues, its expression is largely limited to mesothelial cells, making it a suitable target for tumor-specific therapies.

^90^Y-mAb 22A31 showed significant therapeutic effects in both flank and pleural disseminated tumor models despite using MSTO-211H with low C-ERC/mesothelin expression levels. These findings indicate that C-ERC/mesothelin is a feasible target molecule for RIT-targeting mesothelioma and that radiolabeled mAb 22A31 has potential as an RIT agent. It has been reported that mAb 22A31 alone does not inhibit cell proliferation in vitro. Nonetheless, repeated intratumoral injections exhibit therapeutic efficacy in tumor-bearing mice, while intraperitoneal administration fails to produce such effects in vivo [16]. Therefore, the observed therapeutic effect is most likely attributable to the cytotoxic radiation emitted by the radionuclide. Since the relatively slow blood clearance was observed in the ^111^In-mAb 22A31 biodistribution study, the maximum dose of ^90^Y-mAb 22A31 was set to 5.55 MBq for the subcutaneous model based on our previous studies [17,18]. As there are no reports regarding intrapleural administration, the dose was set to 3.7 MBq for the peritoneal seeding model to be safe. A higher dose for the peritoneal seeding model may give a better outcome.

In this study, ^90^Y was selected as a radionuclide considering its use in clinical RIT and the fact that the radionuclide is less likely to be released from the antibody in the body compared with radioiodine. Other metal radionuclides, such as lutetium-177 and actinium-225 (^225^Ac), an alpha emitter, would be applied without any difficulty. Since therapeutic effects have been achieved with intrapleural administration in a pleural dissemination model, it is possible to achieve an even more effective treatment using short-half-life alpha-emitters such as astatine-211, lead-212, and bismuth-213, which have garnered attention in recent years. It has been reported that intraperitoneal administration results in higher tumor accumulation than intravenous administration in a mouse peritoneal seeding model [19], suggesting that RIT via intrathoracic administration has significant potential. Further studies were required to evaluate the usefulness of RIT with intrathoracic administration.

Several reports have been published on the use of RIT for mesotheliomas. The first-in-human study of a thorium-227-labeled mesothelin-targeting antibody, anetumab, has already been performed [20]. Anetumab was developed for ADC, and a clinical trial has been performed [21]. But it has not been approved yet, and whether properties of anetumab are suitable for RIT remains unclear. Since mAb 22A31 showed high potential for RIT, it is still too early to withdraw from development. Studies will be conducted using alpha-emitters, and if promising results are obtained, the study will proceed to physician-initiated clinical trials. Considering immunogenicity, humanization of the antibody may also need to be considered. As another target molecule, RIT targeting of podoplanin has been actively investigated [22,23]. Podoplanin, similar to mesothelin, is expressed in most cases. Although it is difficult to compare the results due to differences in the tumor models, the therapeutic effect of ^90^Y-mAb 22A31 appears to be largely consistent with the results of ^90^Y-labeled anti-podoplanin antibody. Conversely, ^225^Ac-labeled one showed remarkably high therapeutic efficacy, providing a strong rationale for investigating alpha-emitters. Since the prevalence of expression varies between cases, it is preferable to use mesothelin- and podoplanin-targeted RIT, not exclusively but rather complementarily, tailored to each individual case. Imaging rather than biopsy is useful for selecting suitable patients for each RIT since antigen expression in cancer is heterogeneous. While immunoPET can be performed using the same antibody for diagnosis [24], it would be preferable to develop a low-molecular-weight tracer for PET since immunoPET is costly due to the use of antibodies and is therefore not suitable for patient selection purposes.

This study had some limitations. First, MSTO-211H cells, which express C-ERC/mesothelin at lower levels, were used for the in vivo studies. Ideally, a cell line with high C-ERC/mesothelin expression should have been used to demonstrate greater tumor accumulation and therapeutic efficacy. However, although MSTO-211H cells were used, it is believed that their utility in vivo was sufficiently demonstrated. Second, because normal MSTO-211H cells were used, tumor growth in the peritoneal seeding model could not be monitored. However, because all the mice died after a certain period, a tumor-bearing model was successfully established. These results were considered reliable because a significant therapeutic effect was observed. Third, because the availability of mAb 22A31 was limited, it was difficult to perform in vivo blocking studies using unlabeled antibodies or to evaluate the therapeutic effect of the antibody itself. However, the specificity for mesothelin has been demonstrated in vitro, and the therapeutic effect of mAb 22A31 is expected to be minimal in this mouse model. Due to the limited amount of antibody, hematological or histopathological toxicity was not evaluated. For the same reason, the number of DTPA molecules bound per antibody was not measured. However, only five DTPA equivalents were added, and based on experience, it is expected to be 1–2 units, so it should not affect the results this time. Due to restrictions on the use of ^90^Y, therapeutic experiments using non-specific antibodies have not been conducted. Further research is warranted to clarify these findings.

## 4. Materials and Methods

### 4.1. General

The anti-C-ERC/mesothelin antibody (mAb 22A31) was purchased from ImmunoBiological Laboratories (Fujioka, Japan). The human malignant mesothelioma cell lines MSTO-211H, NCI-H28, and NCI-H2052 were purchased from the Japanese Cancer Research Resources Bank (Tokyo, Japan).

### 4.2. Radiolabeling Antibody

^125^I-labeled mAb 22A31 was prepared using the chloramine-T method with small-scale [25]. Briefly, 2 µL (740 kBq) of Na^125^I solution (GE Healthcare, Buckinghamshire, UK) and 3 µL of chloramine-T solution (1 µg) in 0.3 M phosphate buffer were added to 10 µg of mAb 22A31 dissolved in 100 µL of 0.3 M phosphate buffer. After incubation at room temperature for 5 min, the labeled antibody was purified using a gel filtration column, Bio-spin 6 Column (Bio-Rad, Hercules, CA, USA), according to the manufacturer’s protocol, with phosphate-buffered saline (PBS) as the eluent. For labeling with ^111^In or ^90^Y, 2-(4-isothiocyanatobenzyl)-diethylenetriamine pentaacetic acid (SCN-Bn-DTPA; Macrocyclics, Dallas, TX, USA) was conjugated with mAb 22A31. SCN-Bn-DTPA in dimethylformamide was added to the antibody (5–10 mg/mL in 50 mM borate-buffered saline, pH 8.5) at a molar ratio of 5:1. The mixture was incubated at 37 °C for 20 h, and the DTPA-conjugated antibody was purified using a Bio-spin 6 column. The number of DTPA attached per molecule of mAb was not measured, but it is estimated to be 1.0–2.0 based on past experimental results [16,17]. To prepare ^111^In or ^90^Y-labeled antibody, 50 µL (3.7 MBq) of ^111^InCl_3_ solution (Nihon Medi-Physics, Tokyo, Japan) or 40–60 µL (100–150 MBq) of ^90^YCl_3_ solution (nuclitec GmbH, Braunschweig, Germany) was incubated with 100 µL of 0.25 M acetate buffer (pH 5.5) for 5 min at room temperature. This mixture was then added to 300–500 µg of DTPA-antibody in 0.25 M acetate buffer (pH 5.5) and incubated for 1 h at 37 °C. The radioactivity and antibody amount used were determined considering the specific activity of ^90^Y-mAb 22A31 (20 µg/3.7 MBq) for administration in animal experiments. The radiolabeled antibody was purified using a PD-10 column (GE Healthcare), according to the manufacturer’s protocol. The radiochemical purity of the radiolabeled antibody was >95%, determined by Tec-Control Chromatography Strips (Biodex Medical Systems, Shirley, NY, USA) developed with saline.

### 4.3. Western Blot Analysis for C-ERC/Mesothelin Expression

The cells were dissolved in Laemmli sample buffer (31 mg DTT/mL 4 × Laemmli buffer (Bio-Rad Laboratories)). The protein concentration was determined using the Protein Assay BCA kit (Wako, Osaka, Japan). Proteins (50 μg) were fractionated on a 5–20% gradient SDS-PAGE gel (Atto, Tokyo, Japan) and blotted onto a PVDF membrane (Millipore, Billerica, MA, USA) by electrotransfer. After blocking for 1 h, the blots were then incubated overnight at 4 °C with primary antibodies against mAb 22A31 or anti-β-actin (clone AC-15; Sigma-Aldrich, Saint Louis, MO, USA) as a loading control. The bound antibodies were detected using horseradish peroxidase-conjugated secondary antibodies (sc-2030; Santa Cruz Biotechnology, Inc., Santa Cruz, CA, USA) or anti-mouse IgG (KPL Inc., Gaithersburg, MD, USA) developed using Amersham ECL prime detection reagents (GE Healthcare). Membranes were imaged using the ImageQuant™ LAS 4010 (GE Healthcare), and image band intensities were quantified using ImageJ 1.47 software. Data were normalized to the NCI-H28.

### 4.4. Binding Assay

For the cell binding assays, 100 µL of ^125^I-mAb 22A31 in PBS (containing 0.001 µg antibody and 0.1% BSA) was added to 100 µL of each cell suspension of MSTO-211H, NCI-H28, and NCI-H2052 with different cell numbers (1.0 × 10^5^, 1.0 × 10^6^, and 1.0 × 10^7^). After incubation for 1 h at room temperature, the suspensions were centrifuged at 1580× *g* for 5 min. The supernatant was removed, and the radioactivity of the cell pellets was measured using a well-type gamma counter (ARC7001; Hitachi Aloka, Tokyo, Japan). Cell-bound radioactivity was calculated as a percentage of the initial radioactivity added. For competitive inhibition assays, 100 µL of ^125^I-mAb 22A31 in PBS (containing 0.001 µg antibody and 0.1% BSA) was added to 100 µL of NCI-H2052 cell suspension (containing 5 × 10^5^ cells each) with various amounts of nonlabeled mAb 22A31. After incubation for 1 h at room temperature, the cell suspension was centrifuged at 1580× *g* for 5 min. Then, cell-bound radioactivity was calculated as described above. Antigen expression levels (Bmax) and dissociation constant (Kd) of the antibody were determined via Scatchard plot analysis.

### 4.5. Biodistribution Study

Animal studies were performed according to institutional guidelines and approved by the Local Animal Care Committee of Gunma University. BALB/c nude mice were purchased from CLEA Japan (Tokyo, Japan). Tumor-bearing mice were prepared by injection of MSTO-211H tumor cells (2 × 10^6^ cells) in 100 µL PBS into the right flank of each mouse. When the tumors had fully grown, animals were randomly assigned to each treatment group (five mice per time point). A biodistribution study was performed by intravenous administration of ^111^In-mAb 22A31 (protein dose: 15 µg) in 100 µL PBS into the tail vein of mice. At 1, 24, and 48 h after injection, animals were euthanized by decapitation, and the tissues of interest were excised and weighed. Radioactivity was measured using a well-type gamma counter.

### 4.6. Therapeutic Study

For the therapeutic study of the subcutaneous implantation model, mice were prepared as mentioned above. When the tumors had grown sufficiently (average tumor size: 83.8 ± 21.5 mm^3^), animals were randomly assigned to each group (four mice per group). The tumor-bearing mice were intravenously administered 3.7 MBq or 5.55 MBq of ^90^Y-mAb 22A31 (protein dose: approx. 20 µg/3.7 MBq, injection volume: 100 µL/3.7 MBq) in PBS into the tail vein of mice. The untreated group was used as a control. The mice were weighed, and the tumor diameters were measured using calipers twice per week. Tumor volumes were calculated according to the following formula: (length) × (width)^2^ × 0.5, and the relative tumor size was calculated by dividing each initial tumor volume. Mice with a tumor volume > 2000 mm^3^ or reduced activity were euthanized by cervical dislocation.

For the therapeutic studies of the pleural dissemination model, mice were prepared by injection of MSTO-211H tumor cells (2 × 10^6^ cells) in 100 µL PBS into the thoracic cavity of BALB/c nude mice, referencing previous reports [26]. Four weeks after the injection, the animals were randomly assigned to each treatment group (nine mice per group). The tumor-bearing mice were administered 3.7 MBq of ^90^Y-mAb 22A31 (protein dose: approx. 20 µg) in PBS (100 µL) into the thoracic cavity. The untreated group was used as a control. The mice were monitored until their death or a decrease in activity was observed. Mice with reduced activity were euthanized by cervical dislocation.

### 4.7. Statistical Analysis

Data are presented as means ± standard deviation, where appropriate. Statistical analysis was performed using GraphPad Prism version 9.2.0. Significant differences from the control group were analyzed using Dunnett’s test. The cumulative probability of survival was estimated in each group using the Kaplan–Meier survival curve analysis, and the results were compared using the log-rank test. Differences were considered statistically significant at *p* < 0.05. Correlation was considered very strong when the correlation coefficient (r) was more than 0.9.

## 5. Conclusions

Radiolabeled mAb 22A31 specifically bound to C-ERC/mesothelin-positive cell lines and accumulated in tumor-bearing mice. ^90^Y-mAb 22A31 showed significant therapeutic effects in both flank and pleural disseminated tumor models. These findings indicate that C-ERC/mesothelin is a promising target molecule and that radiolabeled mAb 22A31 is a promising agent for RIT in mesothelioma.

## Data Availability

The original contributions presented in this study are included in the article/supplementary material. Further inquiries can be directed to the corresponding author.

## Supplementary Materials

The following supporting information can be downloaded at: https://www.mdpi.com/article/10.3390/ph19030501/s1. Figure S1: Photograph of the pleural cavity 4 weeks after MSTO-211H cell injection. Figure S2: The original Western blotting image shown in Figure 1A.

## Abbreviations

The following abbreviations are used in this manuscript:

RIT	Radioimmunotherapy
ADC	Antibody–drug conjugate
CAR-T	Chimeric antigen receptor T Cells
DTPA	Diethylenetriamine pentaacetic acid
PET	Positron emission tomography
PBS	Phosphate-buffered saline
