# Supplementary material for: Radioimmunotherapy for Malignant Mesothelioma Targeting C-ERC/Mesothelin
Source: Pharmaceuticals (Basel). 2026 Mar 18;19(3):501. doi: 10.3390/ph19030501 (PMC13029053; doi:10.3390/ph19030501)
Supplement: Supplementary file 1 [file pharmaceuticals-19-00501-s001.zip › pharmaceuticals-4093810-supplementary.pdf]

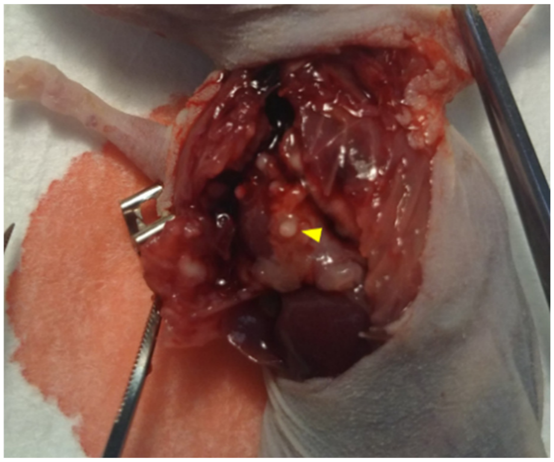

Supplemental Figure S1. Photograph of the pleural cavity 4 weeks after MSTO-211H cell injection. Many pleural dissemination lesions (a small white lump indicated by the yellow arrow head) are observed.

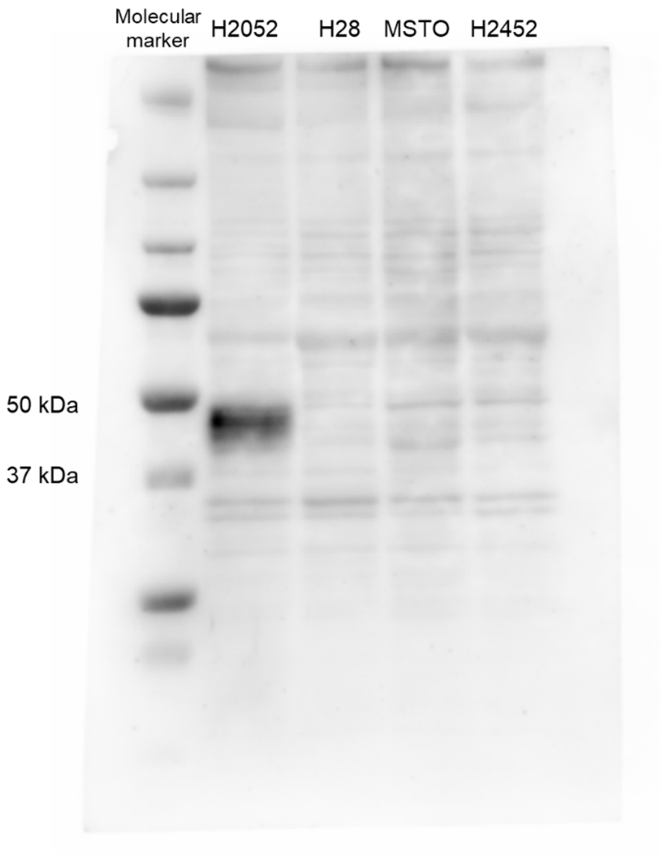

Supplemental Figure S2. The original western blotting image shown in Figure 1A. H2052: NCI-H2052, H28: NCI-H28, MSTO: MSTO-211H. We also analyzed H2452 cells, but did not conduct any further investigation in this study, so we did not include the data in the manuscript.
